# Default and executive networks’ roles in diverse adolescents’ emotionally engaged construals of complex social issues

**DOI:** 10.1093/scan/nsab108

**Published:** 2021-10-01

**Authors:** Rebecca Gotlieb, Xiao-Fei Yang, Mary Helen Immordino-Yang

**Affiliations:** School of Education and Information Studies, University of California, Los Angeles, CA 90095-1521, USA; Center for Affective Neuroscience, Development, Learning and Education, Brain and Creativity Institute, Rossier School of Education, University of Southern California, Los Angeles, CA 90089-2921, USA; Center for Affective Neuroscience, Development, Learning and Education, Brain and Creativity Institute, Rossier School of Education, University of Southern California, Los Angeles, CA 90089-2921, USA; Psychology Department, Neuroscience Graduate Program, University of Southern California, Los Angeles, CA 90089, USA

**Keywords:** people of color, frontoparietal brain network, default mode network, abstract thought, empathy

## Abstract

Across adolescence, individuals enrich their concrete, empathic, context-specific interpretations of social-world happenings with abstract, situation-transcending, system-level considerations—invoking values, bigger implications and broader emotional perspectives. To investigate neural mechanisms involved in abstract construals *vs* concrete construals and the effects of emotional engagement on these mechanisms, 65 mid-adolescents aged 14–18 years reacted to compelling video mini-documentaries during private, open-ended interviews and again during functional magnetic resonance imaging. Following calls to diversify samples, participants were ethnically diverse low-socioeconomic status (SES) urban adolescents performing well in school. Participants spontaneously produced both concrete and abstract construals in the interview, and tendencies to produce each varied independently. As hypothesized, participants who made more abstract construals showed a greater subsequent default mode network (DMN) activity; those who made more concrete construals showed greater executive control network (ECN) activity. Findings were independent of IQ, SES, age and gender. Within individuals, DMN activation, especially when individuals were reporting strong emotional engagement, and ECN deactivation together predicted an abstract construal to a trial. Additionally, brief ECN activation early in the trial strengthened the DMN–abstraction relationship. Findings suggest a neural mechanism for abstract social thought in adolescence. They also link adolescents’ natural construals of social situations to distinct networks’ activity and suggest separable sociocognitive traits that may vary across youths.

## Introduction

During adolescence, youths’ ability to interpret and emotionally react to socially gleaned information becomes increasingly sophisticated. Unlike younger children, mid-adolescents more facilely engage in complex, inferential reasoning about the stories they encounter, and in fact, they are often driven to do so (Erikson, 1968). As they deliberate, they develop skill in appropriately invoking not just concrete interpretations that pertain to the emotional and social happenings directly relevant to a story context, but they also begin to apply abstract thinking, inferring the broader and system-level implications, emotionally poignant values and lessons that transcend the immediate situation (Fischer, 1980; Bruner, 1986/2009; Damon *et al.*, 2003; Steinberg, 2014). For example, an adolescent may react to the story of Malala Yousafzai standing up to the Taliban-enforced education ban for Pakistani girls by expressing his shared sadness and frustration—consistent with a concrete construal and empathic emotion. He may also consider the broader implications for human rights—indicating progression to an abstract construal that may drive strong emotions pertaining to broadly applicable values, such as gratitude for his own education or commitment to social justice. How do adolescents’ newly developing abilities to think abstractly converge with their propensity for strong social emotion, moving them to feel deeply about complex social issues?

To begin to address this question, here we conducted a mixed-method study in which we interviewed 14- to 18-year-old community adolescents from diverse backgrounds about their open-ended interpretations and emotional feelings when reacting to true stories about other adolescents from around the world, presented in the form of mini video documentaries. Participants then underwent functional magnetic resonance imaging (fMRI) while reacting again to the same stories and indicated their current strength of emotional engagement. We sought to understand how adolescents’ natural construals of compelling stories in the interview would predict their neural activity, both across individuals (revealing traits) and within individuals (revealing mechanisms). Because experiencing emotion is physiologically arousing and mentally engaging (Carvalho and Damasio, 2021), we also sought to understand how emotional engagement would influence network activity. Consistent with calls to broaden representation in psychological and neurodevelopmental research (Rad *et al.*, 2018; Buchanan *et al.*, 2020; Qu *et al.*, 2021), participants were diverse youth of color in good academic and disciplinary standing at high schools in low-SES urban communities.

Two networks we hypothesized would be involved in forming abstract construals, as compared to concrete construals, are the default mode network (DMN) and executive control network (ECN), both of which undergo extensive development across adolescence (Fair *et al.*, 2009; Zielinski *et al.*, 2010; Jolles *et al.*, 2011; Raznahan *et al.*, 2011; Satterthwaite *et al.*, 2013; Sherman *et al.*, 2014). Abstract cognition involves transcending the immediate environment, gating available perceptual information and decreasing motor control, in the service of internal generation of a narrative (Liberman and Trope, 2008; Immordino-Yang, 2016; Yang *et al.*, 2018). Previous work has shown that abstract construals of stories are facilitated when individuals avert their gaze or close their eyes and that this gaze aversion predicts subsequent DMN activity (Yang *et al.*, 2018). The DMN is believed to be involved in internally generated thoughts including imagining, thinking about oneself or others and prospecting (Greicius *et al.*, 2003; Andrews-Hanna *et al.*, 2010; Spreng and Grady, 2010; Raichle, 2015; Mak *et al.*, 2017). The core of the DMN, which is centered in the anterior medial prefrontal cortex and inferior-posterior sector of posteromedial cortices (including posterior cingulate cortex and inferior precuneus; Parvizi *et al.*, 2006), is involved in integrating information to build personal meaning and self-understanding, evaluating the personal significance of information and making affectively informed social decisions (Andrews-Hanna *et al.*, 2010). Consistent with its role in the subjective, personal process of reflecting, reasoning and making social-psychological and moral inferences (Immordino-Yang *et al.*, 2012; Dumontheil, 2014; Mooneyham *et al.*, 2016; Tamir *et al.*, 2016; Kaplan *et al.*, 2017), the DMN has been specifically implicated in early adulthood in the formation of abstract construals of stories invoking admiration and compassion (Immordino-Yang *et al.*, 2009; Yang *et al.*, 2018), and its maturation has been related to adolescents’ comprehension of story narratives (Horowitz-Kraus *et al.*, 2017). Given this, we hypothesized that neural regions associated with the DMN would be relatively more active across the experiment in adolescents who utilized abstract construals more often in the interview (but not regions associated with the ECN; Hypothesis 1a) and among participants who reported stronger emotional engagement (Hypothesis 1b).

The ECN (alternatively referred to as the central executive network, the executive attention network or the frontoparietal network) is composed of regions in the dorsolateral prefrontal cortices, dorsomedial prefrontal cortex, inferior parietal lobule, anterior–superior posteromedial cortices and medial temporal gyrus (Seeley *et al.*, 2007; Barrett and Satpute, 2013; Spreng *et al.*, 2013; Dixon *et al.*, 2018). It supports goal-directed actions, focused attention, inhibition, working memory and contextualized cognition (Seeley *et al.*, 2007; Sridharan *et al.*, 2008; Barrett and Satpute, 2013; Satterthwaite *et al.*, 2013; Spreng *et al.*, 2013). In essence, the ECN is functionally involved in thinking and acting in the here-and-now (Immordino-Yang *et al.*, 2012), in regulating emotional reactions (Etkin *et al.*, 2015) and in marshaling one’s attentional focus (Dixon *et al.*, 2018). Unlike abstract construals, concrete construals pertain to perceptually salient information discernable from the current context (Liberman and Trope, 2008). Given this, we hypothesized that neural regions associated with the ECN would be relatively more active across the experiment in participants who utilized concrete construals more often in the interview (but not regions associated with the DMN; Hypothesis 2a) and who reported stronger emotional engagement (Hypothesis 2b).

Following our interest in elucidating the mechanism underlying abstract construals in adolescents, we also tested the contributions of DMN and ECN neural activity and of emotional engagement to predicting abstract construals trial-by-trial. We hypothesized that the DMN, but not ECN, would be differentially activated in scanner trials corresponding to a story stimulus in which the participant’s interview response had contained abstract construals and that this effect would be strengthened when the participant reported stronger emotional engagement; parallel effects would not hold for concrete construals (Hypothesis 3). We were also interested to discover whether ECN activity would contribute to explaining abstract construals trial-by-trial. Abstract construals are just developing and potentially effortful among adolescents, and recent research suggests that the ECN and DMN may coordinate or briefly coactivate under conditions requiring focused attention to generate an insight or divergent idea that is novel, complex, creative or artistic (Christoff *et al.*, 2009; Spreng and Grady, 2010; Smallwood *et al.*, 2012; Beaty *et al.*, 2015, 2016, 2018; Dixon *et al.*, 2018). It may be that coordinated ECN activity constrains free-form daydreaming and protects against distraction, setting the stage for the DMN to process conceptually complex abstract thoughts (Christoff *et al.*, 2009; Smallwood *et al.*, 2012), including those with strong emotional implications (Immordino-Yang *et al.*, 2012). As such, although we did not expect greater ECN activity across a trial to be associated with abstraction, we tested the possibility that early in the trial an increase in ECN activity would be associated with a stronger relationship between DMN activity and abstraction (Hypothesis 4). Such a finding would strengthen the interpretation that these networks’ cooperation is important for complex, emotionally engaged thinking.

Given that abstract construals rely on actively developing cognitive capacities that may vary across youths’ developmental contexts, we examined and accounted for participants’ IQ, gender, age and family socioeconomic status (SES; indexed by free/reduced school lunch, a measure of family financial income to need ratio and parental education level).

## Methods

These data were collected as part of a larger longitudinal project for which participants also completed activities unrelated to the present study (e.g., resting-state fMRI and interviews about school). See SI.

### Participants

We recruited a community sample of 65 youths (36 reporting to be females; mean age = 15.77, s.d.* *= 1.05, range 14–18 years old) from local high schools serving low-SES neighborhoods in Los Angeles. Participants and their parents gave written informed consent, in accordance with the requirements of the Institutional Review Board of the University of Southern California. All participants were performing well in schools based on parent and counselor reports, not under disciplinary action, reported no drug or alcohol use, reported no neurological or psychiatric issues and reported being right-handed. Fifty-one reported participating in the school free/reduced-price lunch program (a measure of family income/needs, an effective proxy for SES; Nicholson *et al.*, 2014). Parental education, an additional proxy for SES, ranged from 8 to 18 years (*M *= 12.34, s.d.* *= 3.85). Thirty-four participants identified as Latinx, 29 as East Asian American and 2 as African American. Participants’ IQs were in the normal range based on Matrix Reasoning and Vocabulary sub-scales of the Wechsler Abbreviated Scale of Intelligence—second edition (Wechsler, 2013) [*M* = 104.52 (s.d. = 11.51), median 105, range 79–131] (see SI). We were unable to use neuroimaging data from one participant due to excessive sleepiness during fMRI scanning. No additional participants were excluded.

### Procedure

Adapting an established protocol (Immordino-Yang *et al.*, 2009), participants completed a private 2 h, videotaped interview during which an experimenter shared 40 compelling true stories about living, non-famous adolescents from around the world. Stories followed a consistent format, beginning with ‘This is a story about a boy/girl …’ followed by the experimenter telling a memorized scripted story and showing an ∼1 min documentary-style video featuring the protagonist (not an actor). The experimenter then asked, ‘How does this story make you feel?’. Participants were encouraged to be as candid as possible. The interview was videotaped, and the experimenter recorded verbatim responses by hand-written notes.

To induce varied emotional reactions and construals and maintain participants’ interest, we included a range of compelling stories in four categories previously piloted/utilized to invoke primarily positive or negative emotion and primarily concrete or abstract construals (Immordino-Yang *et al.*, 2009; Yang *et al.*, 2018). Stories were presented during the interview in one of two pseudo-random counterbalanced orders, with no more than two stories from the same category presented in a row.

After the interview and a short snack/bathroom break, participants completed a blood oxygen level–dependent (BOLD) fMRI scan. During scanning, participants watched a 5 s reminder clip depicting the crux of each story stimulus from the interview, accompanied by one summary sentence presented in both auditory and written forms. Each clip was followed by a 13 s gray screen and then a 2–6 s fixation cross (to jitter the interstimulus interval). At any time during the video or gray screen, participants indicated by pushing a button resting in their right hand the strength of emotional engagement they were currently experiencing, from not particularly emotional (pointer finger), to moderately emotional (middle finger), to very strongly emotional (ring finger) to overwhelmingly emotional (pinky finger). Button presses were recorded numerically as 1–4. Post-scanning, participants were debriefed to confirm that they had thought and felt about the stories similarly to how they had during the interview.

Stories were presented in the scanner in one of two counterbalanced fixed pseudo-random orders. Each story was presented twice over the course of the experiment but never twice in the same run. This resulted in 80 story presentations divided into four runs of ∼7 min each. Data from the two scanner presentations of the same story were averaged to produce one scanner ‘trial’.

#### MRI data acquisition

BOLD fMRI scanning was conducted with a 3T Siemens Trio scanner with a 12-channel matrix head coil. Four runs of functional scans with 233 volumes each were acquired using a T2*-weighted echo-planar imaging sequence (TR = 2 s, TE = 25 ms, flip angle = 90°, acquisition matrix: 64 × 64, FOV = 192 mm) with a voxel resolution of 3 × 3 × 3 mm. Forty-one continuous transverse slices were acquired in interleaved order to cover the whole brain and brain stem. Anatomical images were acquired using a magnetization-prepared rapid acquisition gradient echo sequence (TI = 800 ms, TR = 2530 ms, TE = 3.09 ms, flip angle = 10°, isotropic voxel resolution of 1 mm, acquisition dimensions: 256 × 256 × 176).

### Neural data processing and analysis

#### fMRI data pre-processing

fMRI neuroimaging data underwent preprocessing using SPM12 and MATLAB 2015b (Wellcome Department of Cognitive Neurology, London, UK; MathWorks, Inc., Natick, MA, USA). Functional images were slice timing and motion corrected and co-registered to the anatomical image. Anatomical images were normalized to the Montreal Neurological Institute (MNI) space using the segmentation procedure. The resulting normalization transformation was applied to the functional images. Data were visually inspected at each step. The functional images were resampled into an isotropic voxel resolution of 2 × 2 × 2 mm and smoothed using an 8 mm full-width at half maximum Gaussian kernel. The Artifact Detection Tool (ART; https://www.nitrc.org/projects/artifact_detect/) was used to identify motion outliers in the functional images for motion scrubbing (frame-to-frame composite movement exceeding 1 mm or normalized frame-to-frame global signal change exceeding a *z*-score of 9). Participants with ≥20% of functional volumes identified by ART as motion outliers, or with average frame-to-frame composite motion exceeding 1 mm, would have been excluded from the analysis. Because no participants met these criteria, none were excluded for motion.

#### Modeling neural responses to story stimuli (whole-brain analyses)

We tested Hypotheses 1a and 2a with whole-brain analyses. At the individual level, following previous work (Immordino-Yang *et al.*, 2009; Yang *et al.*, 2018), each story category was modeled using a finite impulse response function with nine time bins, each corresponding to a 2 s TR. Six motion parameters (three translations and three rotations) calculated during the motion correction procedure, and single-TR spike regressors corresponding to the identified motion outliers, were included as covariates of no interest. The inclusion of spike regressors excluded motion outlier volumes from the analysis. To characterize the level of activity, contrast maps for story trials *vs* implicit baseline were calculated for each story category by averaging parameter estimates over the 4th–8th time bins [corresponding to the 6–16 s post-stimulus onset or ∼2–12 s, adjusting for hemodynamic delay, the time window previously identified as appropriate for this task (Immordino-Yang *et al.*, 2009)].

At the group level, contrast maps were entered into a whole-brain multiple regression model with summed concrete and abstract construal scores as covariates. Given known neural activation and construal differences across the story categories in adults (Immordino-Yang *et al.*, 2009; Yang *et al.*, 2018), we controlled for the main effect of the story category. To isolate the effect of construals from the effects of covariates, we repeated the whole-brain analysis controlling for age, gender, SES (lunch status and parental education) and IQ.

The results were subjected to a cluster forming threshold of *P* < 0.001, and a cluster extent threshold of 219 voxels, which controls the probability of false positives at <5%. The cluster extent threshold was determined by 10 000 Monte Carlo simulation iterations conducted using the 3dClustSim program in AFNI (version 21.0.21; http://afni.nimh.nih.gov/afni/). The criteria input to 3dClustSim were: cluster forming threshold of *P* = 0.001, voxel size of 2 × 2 × 2, the spatial auto-correlation function estimated from the model residual maps using 3dFWHMx in AFNI and whole-brain analysis mask (containing 174 671 voxels).

#### Identification of regions of interest

**Fig. 1.**
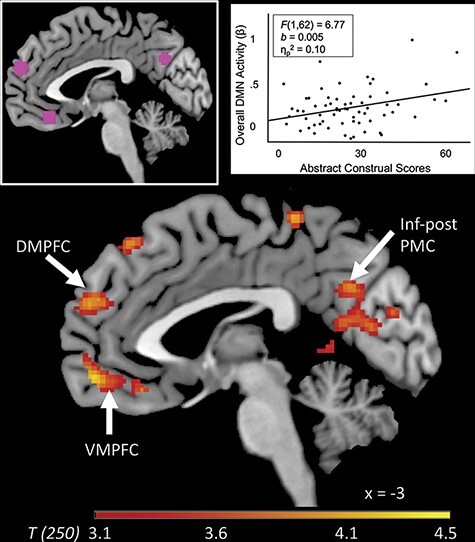
Neural correlates of abstract construals. Results from a whole-brain analysis reveal regions whose activity while responding to documentary-style stories positively correlates with abstract construal scores from the interview (*N* = 64). The image is subjected to a cluster forming threshold of *P* < 0.001, and cluster extent thresholded at *k* = 177 voxels (for illustrative purposes). The in-set image depicted in purple correspond to 6 mm spherical ROIs located in the DMN. The in-set scatterplot depicts participants’ average parameter estimates (β) from all voxels within the identified ROIs relative to abstract construal scores. Each dot represents one participant. Inf-post PMC = inferior/posterior posteromedial cortices; DMPFC = dorsomedial prefrontal cortex; VMPFC = ventromedial prefrontal cortex.

**Fig. 2.**
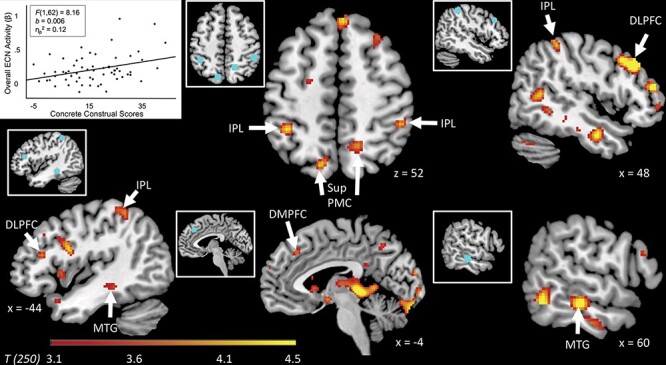
Neural correlates of concrete construals. Results from a whole-brain analysis reveal regions whose activity while participants responded to documentary-style stories positively correlates with concrete construal scores from the interview (*N* = 64). Images are subjected to a cluster forming threshold of *P* < 0.001, and cluster extent thresholded at *k* = 16 voxels (for illustrative purposes). The in-set images depicted in blue correspond to 6 mm spherical ROIs located in the ECN. The in-set scatterplot depicts participants’ average parameter estimates (β) from all voxels within the identified ROIs relative to concrete construal scores. Each dot represents one participant. Ant-Sup PMC = anterior/superior posteromedial cortices; DMPFC = dorsomedial prefrontal cortex; DLPFC = dorsolateral prefrontal cortex; IPL = inferior parietal lobule; MTG = medial temporal gyrus.

Key regions of the DMN and ECN were identified based on the visual inspection of positive results from the whole-brain voxel-wise analysis. Regions of interest (ROIs) corresponding to key hubs of the DMN and ECN were functionally defined as 6 mm spheres centered at the local maximum within the relevant cluster. (See inset images in Figures 1 and 2 and bolded clusters in Table 1.) Five clusters that met the cluster forming threshold but not the cluster extent threshold were included in our ROIs because these fell in canonical regions associated with the DMN (dorsomedial prefrontal cortex; Andrews-Hanna *et al.*, 2010) and the ECN (left medial temporal gyrus, dorsomedial prefrontal cortex, bilateral inferior parietal lobule; Seeley *et al.*, 2007).

**Table 1.** Peak coordinates in MNI space of clusters identified via whole-brain voxel-wise analysis, whose BOLD activity positively correlates with: A. abstract construal scores, or B. concrete construal scores

	Coordinate				
Region	*X*	*Y*	*Z*	*z*-Score	Cluster size
**A. Neural correlates of abstract construals**					
Dorsomedial prefrontal cortex	−4	60	22	4.03	177†
	**−2**	**58**	**24**	3.94	
Ventromedial prefrontal cortex	−4	56	−12	4.21	290*
	**−4**	**34**	**−16**	4.16	
Inferior–posterior posteromedial cortices	12	−96	8	6.58	1142**
	**2**	**−64**	**32**	4.54	
Pre-supplementary motor area (left)	−16	30	58	6.25	777**
**B. Neural correlates of concrete construals**					
Inferior frontal gyrus	−52	42	−8	4.47	283*
Dorsolateral prefrontal cortex (left)	**−40**	**30**	**18**	4.15	
Dorsolateral prefrontal cortex (right)	**44**	**28**	**34**	5.78	1230**
Medial temporal gyrus (left)	**−44**	**−34**	**−10**	3.44	16†
Inferior temporal gyrus	56	−50	−14	5.31	1764**
Medial temporal gyrus (right)	**60**	**−24**	**−12**	4.77	
Superior posteromedial cortices (left)	**−12**	**−72**	**54**	4.21	
Superior posteromedial cortices (right)	**16**	**−56**	**50**	4.07	
Dorsomedial prefrontal cortex	**−4**	**34**	**40**	3.61	37†
Inferior parietal lobule (left)	**−40**	**−44**	**56**	4.75	174†
Inferior parietal lobule (right)	**48**	**−40**	**52**	4.02	52†
Cerebellum (right)	30	−88	−22	4.89	515**
White matter (left)	−24	−36	42	4.51	315*
Cerebellum (right)	30	−44	−24	4.24	221*
Brainstem	−2	−36	−2	5.24	878**
Midbrain	12	−6	−18	5.11	737**

Bold coordinates represent peaks that match the known executive control network or default mode network hubs, used to construct network ROIs (ROIs are depicted in Figures 1 and 2).

Greatest local maximum is reported for each cluster. For larger clusters that extend into multiple anatomical regions, other notable local maxima are reported (indented). Clusters are significant at *P* < 0.05, corrected for multiple comparisons (corresponding to a cluster forming threshold of *P* < 0.001 and a cluster extent threshold of 219 voxels), except those labeled †, which are included because they fall in canonical DMN and ECN regions. Regions not labeled left or right extend bilaterally.

** *P* < 0.01,

* *P* < 0.05,

† not significant.

#### Calculating neural network activity for trait-level analyses

The MarsBar toolbox was used to calculate the average parameter estimates from all voxels within each of the identified ROIs, with activation levels for each story category averaged. The nine ECN ROIs were all positively and significantly correlated, as were the three DMN ROIs (ECN: all *P*’s < 0.002, all *r*’s > 0.41; DMN: all *P*’s < 0.001, all *r*’s > 0.55). We tested all trait-level models with each ROI separately and found the direction of results consistent (see SI) and confirmed that effects were not driven by outliers. Therefore, ROI values corresponding to each network were averaged.

#### Calculating neural network activity for trial-by-trial analyses

Using the MarsBar toolbox, finite impulse response event-related time courses corresponding to the average of the two presentations of the same story stimulus (a neural ‘trial’) were calculated for each ROI. To test Hypothesis 3, TRs 4–8 were averaged, producing one value per story per participant per ROI. To test Hypothesis 4, TRs 4–5 were averaged, producing a value for just the early part of the trial. ROI values corresponding to each network were averaged. Any stimulus presentation containing a motion outlier was excluded, and the data from the remaining story presentation were utilized; all participants had at least 33 usable trials. To isolate within-person variability, DMN and ECN activity scores were mean-centered within each participant; activation for each trial was calculated relative to a participant’s own mean.

### Behavioral data processing and analysis

#### Concrete and abstract construal scores

Videotaped interviews were electronically transcribed verbatim and verified by research assistants who were blind to the study hypotheses.

Transcribed responses were coded by at least two independent raters for concrete and abstract construals. Responses to any particular story could evince multiple codes. Interrater reliability was 98%; discrepancies were resolved by discussion.

Concrete construals were identified as those mentioning empathic emotions (e.g. ‘I just feel, like, so bad for her’) or emotions directly relevant to the participant’s reaction to the protagonist’s current situation (e.g. ‘it makes me happy’), positive comments concerning the protagonist’s actions and choices (e.g. ‘I’m glad she did something like that’), or criticisms of the protagonist’s actions or choices (e.g. ‘I think it was kind of dumb of him. It was kind of his fault’). To accommodate their anti-social and unempathic flavor, concrete construals that criticize the protagonist and indicate a lack of empathy were assigned a value of −1; all other concrete construals were assigned a value of 1.

Abstract construals were identified as those mentioning emotions or questions pertaining to the broader implications or perspectives derived from the story, e.g. ‘it makes me happy for humanity’, or statements reflecting on a broad truth, lesson or value, e.g. ‘I think back to the idea that because children are the future […] we have to be able to inspire people who are growing and have the potential to improve the societies…’. To accommodate their anti-social flavor, abstract construals that dismiss the importance of the situation would have been assigned a −1, although none were made. All other abstract construals were assigned a value of 1.

Codes from each participant’s response to each story in the interview were summed to create a concrete and an abstract score for each story response and an overall score across the experiment. Mean-centered abstract and concrete scores for each story were then calculated. (For 9 participants, sound recording malfunctioned and verbatim, handwritten notes from the interview were used to code participants’ responses; for 10 responses distributed across 6 of these participants, these notes were insufficiently complete to definitively code and the participant’s mean score for that story category was used to compute the overall construal scores. These 10 trials were excluded from trial-by-trial analyses.)

#### Emotional engagement scores

Button press values from each participant’s response to each presentation of each story in the scanner were averaged to create a rating of emotional engagement for each trial and an overall average across the experiment. Mean-centered emotional engagement scores for each trial were calculated.

### Testing trial-by-trial relationships with nested data

To test Hypotheses 3 and 4, we used linear generalized estimating equation (GEE) models (IBM SPSS 25, SPSS Inc., Chicago, IL, USA) with within-person mean-centered neural activity, within-person mean-centered abstract construals and within-person mean-centered emotional engagement nested within participants. In all models, within-person mean-centered abstract construals were the outcome measure. GEE models account for within-participant correlations of repeated measures responses (Ballinger, 2004), and mean-centered values isolate within-participant variation. Following Pekár and Brabec (2018), all models utilized an independent working correlation matrix structure, since trials are not independent but their dependence cannot be specified. As described above, data from the two scanner presentations of the same story were averaged to produce one ‘trial’.

## Results

### Abstract and concrete construals and behavioral results

Of 2590 trials, 1110 (43%) included at least one concrete construal and 1256 (48%) included at least one abstract construal. All participants produced both types of construals across the experiment, and there was considerable variability across participants in construal scores [concrete: *M* = 17.78 (s.d. = 11.45), median 17, range −5, 44; abstract: *M* = 25.14 (s.d. = 14.00), median 24.00, range 2, 64].

Concrete and abstract construal scores were unrelated across the experiment, *r*(63) *= *−0.13, *P = *0.32. Participants tended to construe either concretely or abstractly for any particular trial; however, trial-by-trial, concrete and abstract construals were inversely related, controlling for within-subject effects, Wald χ^2^(1, *N* = 2590 interview trials, nested in 65 participants) = 52.35, *b* = −0.18, SE = 0.03, 95% CI [−0.23, −0.13], *P* < 0.001.

Pearson' correlations revealed that concrete construals were negatively correlated with IQ, *r*(59) = −0.27, *P *= 0.04, while abstract construals were marginally positively correlated with IQ, *r*(59) = 0.21, *P* = 0.11. Spearman correlations revealed that construals were unrelated to SES (family income/needs; parental education; all *P*’s > 0.28; all *r*’s between −0.14 and −0.07). Concrete construals were more common in female than in male participants in this sample, *t*(63) = −3.10, *P* = 0.003, *d* = 0.77; no gender differences were found for abstract construals, *P* = 0.75. Concrete construal score was unrelated to age, *r*(63) = 0.11, *P* = 0.40; abstract construal score was marginally positively related to age, *r*(63) = 0.22, *P *= 0.08.

Participants reported varying levels of emotional engagement with the stories [*M* = 2.20 on a scale from 1 to 4 (s.d. = 0.41), median 2.18, range 1.24–3.36]. Pearson correlations revealed that concrete construals were not systematically associated with emotional engagement, *r*(62) = 0.19, *P* = 0.14, while abstract construals were positively associated, *r*(62) = 0.41, *P* = 0.001. The mean of individuals’ button press response time was 5.20 s (s.d. = 1.55), and every participants’ mean response time fell within the window modeled in neural analyses.

### Trait-level neural results

#### A tendency to make abstract construals was positively related to DMN activity, especially when accompanied by strong emotional engagement

Consistent with Hypothesis 1a, analyses revealed increased neural responses within established DMN regions (but not within ECN regions) as a function of the extent to which individuals engaged in abstract construals (Figure 1 and Table 1A). We repeated the whole-brain analyses controlling for age, gender, SES and IQ (simultaneously), and the results remain consistent.

Consistent with Hypothesis 1b, the reported strength of emotional engagement was associated with increased activity in DMN regions, *F*(1,62) = 5.17, *b* = 0.16, SE = 0.07, 95% CI [0.02, 0.30], *P* = 0.03, η_p_^2^ = 0.08. This relationship holds controlling for age, gender, SES and IQ (simultaneously), *P* = 0.01. Feeling stronger emotion strengthened the relation between abstract construals and DMN activity, *F*(1,60) = 6.98, *b* = 0.01, SE = 0.004, 95% CI [0.003, 0.02], *P* = 0.01, η_p_^2^ = 0.10.

We found no main effects of age, gender, SES or IQ on DMN activations using ROI-based analyses; all *P*’s *> *0.57.

#### A tendency to make concrete construals was positively related to ECN activity

Consistent with Hypothesis 2a, analyses revealed increased neural responses within established ECN regions (but not within DMN regions) as a function of concrete construal scores (for whole-brain voxel-wise results, see Figure 2 and Table 1B). We repeated the whole-brain analyses controlling for age, gender, SES and IQ (simultaneously), and the results remain consistent.

Consistent with Hypothesis 2b, the reported strength of emotional engagement was also related to increased ECN activity, *F*(1,62) = 6.29, *b* = 0.14, SE = 0.06, 95% CI [0.03, 0.26], *P* = 0.02, η_p_^2^ = 0.09. This relationship holds controlling for age, gender, SES and IQ (simultaneously), *P* = 0.02. Feeling stronger emotion did not impact the relation between concrete construals and ECN activity, *P* = 0.14.

We found no main effects of age, gender, SES or IQ on ECN activation using ROI-based analyses; all *P*’s *> *0.11.

### Trial-by-trial, within-person mechanisms underlying abstract construals

We tested Hypothesis 3 with mean-centered values of DMN activity, ECN activity, emotional engagement and the interaction of DMN activity and emotional engagement in one GEE model: Consistent with the hypothesis, abstract construals were associated with greater activity in the DMN and with emotional engagement, Wald χ^2^[1, *N* = 2512 interview responses, nested in 64 participants] = 9.04, *b* = 0.17, SE = 0.06, 95% CI [0.06, 0.29], *P* = 0.003 (DMN); Wald χ^2^[1, *N* = 2512] = 37.56, *b* = 0.13, SE = 0.02, 95% CI [0.09, 0.17], *P* < 0.001 (emotional engagement). The effect of DMN activity on abstract construals was strengthened on trials when the participants reported stronger emotional engagement, Wald χ^2^[1, *N* = 2512] = 4.31, *b* = 0.12, SE = 0.06, 95% CI [0.01, 0.22], *P* = 0.04. Abstract construals were associated with less activity in the ECN, Wald χ^2^[1, *N* = 2512] = 16.84, *b* = −0.33, SE = 0.08, 95% CI [−0.48, −0.17], *P* < 0.001. In a similar model exchanging only the network with which emotional engagement interacts, emotional engagement did not moderate the relationship between ECN activity and abstract construals, *P* = 0.44.

We first tested Hypothesis 4 by adding the interaction of DMN and ECN activity to the model tested in Hypothesis 3. We then tested the impact of ECN on the relationship between DMN activity and abstraction only in the TRs early in the trial (TRs 4–5), in a model with DMN activity, ECN activity and their interaction. The results support the hypothesis: Although ECN activity was not associated with a stronger relationship between DMN activity and abstraction across TRs 4–8 of the trial, *P* = 0.55, increased ECN activity was associated with a stronger relationship between DMN activity and abstraction early in the trial, Wald χ^2^[1, *N* = 2512] = 4.17, *b* = 0.08, SE = 0.04, 95% CI [0.003, 0.16], *P* = 0.04.

## Discussion

The proclivity to think and feel deeply about complex social issues is a hallmark achievement of adolescence. To engage in such abstraction involves perceiving but then transcending the immediate facts and perceptions of a situation to imagine the broader values, multiple perspectives and system-level implications that pertain. We interviewed a community sample of diverse adolescents about true stories and found that all youth in our sample made at least some abstract construals as they explained their reactions. Across the experiment, participants’ tendencies toward making concrete and abstract construals were unrelated and associated with distinct neural network activation patterns, suggesting that these construals reflect independent sociocognitive traits. As hypothesized, participants who made more abstract construals overall showed greater subsequent DMN activity when reacting again to the stories in the fMRI scanner; those who made more concrete construals showed greater ECN activity. Findings were independent of IQ, SES, age and gender, suggesting that these proclivities exist across developmental profiles.

**Fig. 3.**
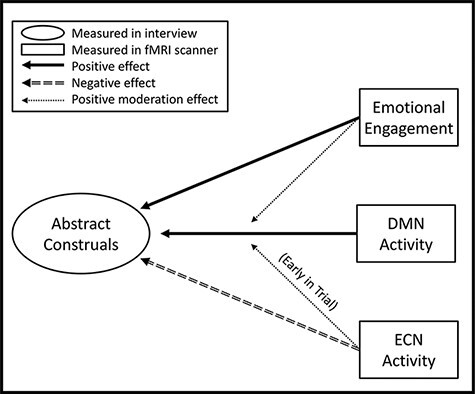
Schematic depicting the trial-by-trial neuropsychological correlates of adolescents’ abstract construals of social stories. Arrows represent statistical analyses for Hypotheses 3 and 4.

Within-person effects, trial-by-trial, also offer evidence of a mechanism for abstract social thought (Figure 3). When adolescents construed a story abstractly, they showed increased subsequent DMN activity when thinking about that story during fMRI scanning, especially if they reported feeling strongly emotionally engaged with the story, and decreased ECN activity. Although construing a story abstractly was associated with decreased ECN activity overall, early in a trial, brief ECN activity strengthened the trial-by-trial relationship between DMN activity and abstraction.

Our study suggests coordinated roles for the DMN and ECN in abstract thinking, consistent with previous theoretical and empirical work (Christoff *et al.*, 2009; Spreng and Grady, 2010; Smallwood *et al.*, 2012; Beaty *et al.*, 2015, 2016, 2018; Dixon *et al.*, 2018). It may be that thinking abstractly about complex issues in adolescence requires not only imaginative thoughts but also early in the process requires focused attention to constrain thinking to the issues at hand. Previous research has suggested that the ECN may comprise two sub-systems, one of which systematically co-activates with the DMN in tasks that involve regulation of introspective processes (Dixon *et al.*, 2018). Interestingly, our whole-brain analyses of trait-level neural correlates of abstraction revealed DMN regions but also a region in the ECN subnetwork that has been found to coactivate with the DMN, the pre-supplementary motor area. More generally, our findings affirm previous work suggesting that the ECN is involved in regulation and contextualized cognition (Spreng *et al.*, 2013; Dixon *et al.*, 2018) and that the DMN is involved in complex, abstract, value-oriented thinking (Immordino-Yang *et al.*, 2012), including to emotionally poignant social stories (Immordino-Yang *et al.*, 2009). The trial-by-trial relation between abstraction and DMN activity replicates our earlier findings with young adults (Yang *et al.*, 2018) and extends these findings to adolescents.

Consistent with a century of human developmental work on adolescents’ meaning-making (Dewey, 1933; Bruner, 1986/2009; Perkins, 2014; Nagaoka *et al.*, 2015) and with educational theory informed by social-affective neuroscience (e.g. Immordino‐Yang and Damasio, 2007; Immordino-Yang *et al.*, 2019), our findings also underscore the notion that rather than interfering with complex cognition, emotion in the context of abstract thinking may drive adolescents’ thinking forward (Fischer, 1980). Potentially, adolescents’ emotional reaction to an issue or idea may motivate them to think harder about it; in the other direction, the complex understanding an adolescent builds may help them appreciate the emotional importance of the issue.

Our study has notable strengths, among them the focus on individual variability, the compelling real-world stimuli and the authentic interview process combined with neuroimaging. Another major strength is the inclusion of low-SES youth of color who are underrepresented in research (Qu *et al.*, 2021). Nonetheless, we note limitations. First, since we were interested in eliciting participants’ open-ended, natural responses to rich social stories, the measure of construals had to be collected prior to the neural measure. Although emotional engagement was reported concurrently with neural data, we cannot definitively confirm that participants’ construal to the story in the scanner matched their construal to the story in the interview. In addition, our hypotheses were a priori but not pre-registered. Future research should replicate this work with other samples and investigate the longitudinal neuropsychological development of abstract social thinking, as well as the origins, malleability and behavioral implications of the individual differences we observe. The extent to which these mechanisms are unique to social thinking, or may be observed in other complex or technical domains, would also be pertinent to investigate, especially because of the possible implications for secondary education (Immordino-Yang, 2016). Our study makes a first attempt to elucidate the dynamics of adolescents’ abstract thinking, but much more work is needed.

In conclusion, adolescence is a unique stage, characterized by intense emotions and social learning, in addition to new intellectual capacities to appreciate the bigger implications of social situations and structures. Our study reveals a neural mechanism for these capacities in adolescence and underscores the emotional nature of adolescents’ abstract social thought. Leveraging these capacities, adolescents can be among the most visionary and idealistic citizens, as the world has recently seen with such extraordinary youths as Malala Yousafzai, Greta Thunberg and Emma González. Understanding the neuropsychological development of capacities for abstract construals and the role of emotional engagement in abstract social thinking, as we have begun to do here, will be central to our understanding of the developmental affordances and needed supports of this transitional life stage.

## Data Availability

The data that support the findings of this study are available on request from the corresponding author. The data are not publicly available due to privacy or ethical restrictions.
